# Stereotactic Body Radiotherapy Without Systemic Therapy for Oligometastatic Cancer: A Systematic Review and Meta-Analysis

**DOI:** 10.1001/jamanetworkopen.2025.49685

**Published:** 2025-12-29

**Authors:** Jonas Willmann, Camilla von Wachter, Ronja Zehnder, Edward Christopher Dee, Hanbo Chen, Lizza E. L. Hendriks, Jordi Remon, Sarah Baker, Michael Mayinger, Nicolaus Andratschke, Narek Shaverdian, Daniel R. Gomez, Umberto Ricardi, Robert Olson, Arjun Sahgal, Dirk De Ruysscher, Puneeth Iyengar, Matthias Guckenberger

**Affiliations:** 1 Department of Radiation Oncology, University Hospital Zurich, University of Zurich, Zurich, Switzerland; 2 Department of Medical Physics, Memorial Sloan Kettering Cancer Center, New York, New York; 3 Department of Radiation Oncology, Memorial Sloan Kettering Cancer Center, New York, New York; 4 Department of Radiation Oncology, Sunnybrook Odette Cancer Centre, Toronto, Ontario, Canada; 5 Department of Pulmonary Diseases, GROW-Research Institute for Oncology and Developmental Biology, Maastricht University Medical Center, Maastricht, the Netherlands; 6 Department of Cancer Medicine, Gustave Roussy, Villejuif, France; 7 Department of Surgery, Division of Radiation Oncology, University of British Columbia, British Columbia, Canada; 8 Department of Surgery, Division of Radiation Oncology, BC Cancer and University of British Columbia, Vancouver, Canada; 9 Department of Oncology, University of Turin, Turin, Italy; 10 Department of Radiation Oncology (Maastro Clinic), Research Institute for Oncology and Developmental Biology (GROW), Maastricht University Medical Center, Maastricht, the Netherlands; 11 Department of Radiotherapy, Erasmus MC, Rotterdam, the Netherlands

## Abstract

**Importance:**

Metastasis-directed stereotactic body radiotherapy (SBRT) may be a viable strategy to defer systemic therapy in patients with oligometastatic cancer due to comorbidities, patient preferences, or concerns about adverse effects. To date, the evidence supporting this approach has not been comprehensively assessed.

**Objective:**

To evaluate systemic therapy–free survival (STFS) for different types of oligometastatic cancer after SBRT alone and to quantify adverse events, quality of life, and oncological outcomes.

**Data Sources:**

A systematic search of PubMed and EMBASE was conducted on July 10, 2024, to identify potentially eligible studies published after 2009. Forward and backward citation tracking was performed to identify additional relevant studies.

**Study Selection:**

Studies eligible for inclusion in the systematic review were retrospective or prospective with at least 10 patients who received metastasis-directed SBRT and no up-front systemic therapy for oligometastatic cancer (≤5 metastases) irrespective of primary tumor histology. Required outcomes being reported were STFS, progression-free survival, or overall survival. The subgroup of studies reporting STFS at 1 or 2 years were included in a meta-analysis of these pooled outcomes.

**Data Extraction and Synthesis:**

Two reviewers independently extracted data using predefined forms and followed the Preferred Reporting Items for Systematic Reviews and Meta-Analyses (PRISMA) guidelines. A random-effects model was used for meta-analysis.

**Main Outcomes and Measures:**

The primary outcome of the meta-analysis was the pooled STFS rate at 1 or 2 years.

**Results:**

Of 29 unique studies (2074 unique patients) included in the systematic review, 13 (984 patients) contributed data to the meta-analysis. The pooled 1- or 2-year STFS rate was 69.7% (95% CI, 57.4%-80.9%) across cancer types. Renal cell cancer demonstrated the highest STFS rate (87.0%; 95% CI, 76.2%-95.2%), followed by prostate cancer (78.1%; 95% CI, 67.4%-87.3%), with lower rates in other cancer types. Among 20 studies reporting adverse events, rates of adverse effects of grade 3 or higher were absent in 15 of 19 studies (79%) and ranged from 2 of 103 (1.9%) to 3 of 34 patients (8.8%) among those that observed severe adverse events.

**Conclusions and Relevance:**

In this systematic review and meta-analysis, metastasis-directed SBRT alone was associated with meaningful STFS, particularly in patients with oligometastatic prostate or renal cell cancer, with low risks of treatment-related adverse events. These findings suggest that SBRT alone might be an acceptable option instead of immediate systemic therapy in selected patients with oligometastatic cancer.

## Key Points

**Question:**

What outcomes are associated with metastasis-directed stereotactic body radiotherapy (SBRT) without up-front systemic therapy in patients with oligometastatic cancer?

**Findings:**

In this systematic review and meta-analysis of 2074 unique patients from 29 unique studies, SBRT alone was associated with pooled 1- and-2-year systemic therapy–free survival (STFS) rate of 70% across cancer types. Subgroup analyses revealed the highest STFS rates in renal cell (87%) and prostate cancer (78%), with lower STFS rates in other cancer types.

**Meaning:**

These findings suggest that metastasis-directed SBRT for oligometastatic disease was associated with clinically meaningful STFS, particularly in selected patients with prostate or renal cell cancer.

## Teaser

This systemic review and meta-analysis evaluates outcomes of patients with oligometastatic cancer treated with metastasis-directed stereotactic body radiotherapy without up-front systemic therapy.

## Introduction

Oligometastatic disease is a state of limited metastatic spread, potentially amenable to metastasis-directed local therapies such as stereotactic body radiotherapy (SBRT).^1,2^ Metastasis-directed SBRT is typically combined with systemic therapy, which is the standard-of-care treatment for most metastatic cancers.^3,4,5^ While SBRT ablates visible tumors, systemic therapy may target occult micrometastases, acting synergistically to prolong disease control. However, systemic therapies have distinct adverse effects and contraindications, limiting use in patients with comorbidities or organ dysfunction.^6,7,8,9^

Consequently, metastases-directed SBRT may be considered to delay or defer systemic therapy, serving as an alternative treatment strategy for patients with oligometastatic disease.^10^ There are several reasons why SBRT may be used without systemic therapy in clinical practice. In cases for which systemic treatment options have limited efficacy and an unfavorable safety profile, alternative treatments may be considered. In particular, older or frail patients and those with comorbidities or persistent adverse effects from prior treatments may not tolerate intensive systemic therapies. Other cancers might be relatively indolent, but their systemic treatments are associated with relevant decrements of quality of life (QOL), for example, for prostate cancer and androgen deprivation therapy (ADT). Some patients also choose to forgo systemic therapy for personal reasons, and SBRT may be offered within a shared decision-making framework to provide local disease control.

The potential of metastasis-directed SBRT to delay systemic therapy has never been comprehensively analyzed across different types of oligometastatic cancers. The association of this treatment approach with adverse events and QOL remains poorly understood. Criteria to guide patient selection and postprogression treatments in clinical practice are lacking. Therefore, we conducted a systematic review and meta-analysis evaluating outcomes of patients with oligometastatic cancer treated with metastasis-directed SBRT alone without up-front systemic therapy.

## Methods

### Study Design and Search Strategy

This systematic review and meta-analysis followed the Preferred Reporting Items for Systematic Reviews and Meta-Analyses (PRISMA) guidelines and was registered in PROSPERO (CRD42024580877). The literature search was performed in PubMed and EMBASE on July 10, 2024. Forward and backward citation searching was applied to identify additional relevant studies. The full search strategy is provided in eMethods 1 in Supplement 1. Studies published after January 2009 were included if they investigated metastasis-directed SBRT of oligometastatic cancer (≤5 metastases) irrespective of primary tumor histology, without immediate systemic therapy in all patients or in a defined subgroup. Eligible studies were prospective or retrospective studies with at least 10 patients reporting systemic therapy–free survival (STFS), progression-free survival (PFS), or overall survival and published in English. There were no restrictions regarding the state of oligometastatic disease.^2^ Studies were excluded if a subset of patients received concurrent systemic therapy or non-SBRT metastasis-directed treatments (eg, surgery), and subgroup outcomes for patients treated with SBRT alone were not separately reported. All studies using metastasis-directed SBRT without immediate systemic therapy were eligible for the systematic review. The meta-analysis included only those studies that reported STFS at 1 or 2 years. Sensitivity analyses including prospective-only studies and leave-one-out analysis assessed robustness of pooled estimates. Two reviewers (C.vW. and R.Z.) reviewed studies and extracted data independently; a third (J.W.) resolved conflicts.

### Data Extraction 

Predetermined forms were used for data extraction. Data were extracted for the study characteristics (author, year of publication, design, sample size, and inclusion and exclusion criteria), patient characteristics (primary tumor, location of metastases), postprogression treatment decisions (application of repeat SBRT and criteria for starting systemic therapy), associated factors and outcomes (STFS, PFS, overall survival, adverse effects, and QOL), and the proportion of study participants without metastases at last follow-up.

### Statistical Analysis

All analyses were based on aggregate-level data extracted from published studies. Individual patient-level data were not available. For the meta-analysis, STFS rates at 1 and 2 years were pooled to maximize eligible studies. A weighted random-effects model was used to estimate pooled 1- or 2-year STFS rates for all studies for which the corresponding outcomes were available. All statistical tests were 2 sided, and *P* < .05 was considered statistically significant. Heterogeneity was assessed using the *I*^2^ statistic and the *Q* test. Interpretation followed Cochrane Handbook guidelines, with descriptive thresholds provided in eMethods 2 in Supplement 1. A univariate meta-regression was performed to evaluate the association of cancer subtype with STFS and to quantify the proportion of heterogeneity explained by this variable. Egger tests and funnel plot inspection were performed for publication bias assessment.^11^ Analyses were performed in Rx64, version 4.2.3 (R Project for Statistical Computing) with the metafor package, version 4.6-0. The Newcastle-Ottawa Scale was used for qualitative assessment of the risk of bias for individual studies. Overall scores of 7 to 9 designated a high-quality study with a low risk of bias; 4 to 6, a moderate-quality study with some risk of bias; and 3 or less, a low-quality study with a high risk of bias.

## Results

### Study Characteristics

**Figure 1.**
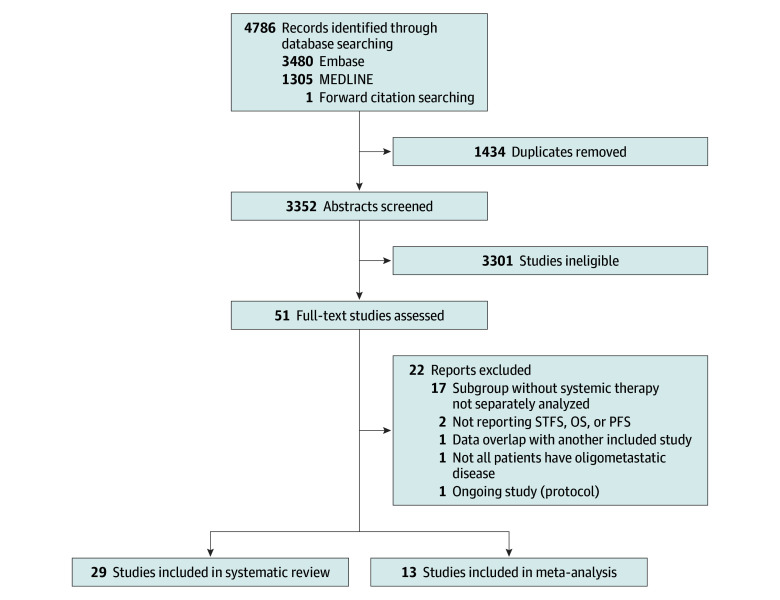
Article Selection Flowchart OS indicates overall survival; PFS, progression-free survival; and STFS, systemic therapy–free survival.

From 3351 records screened, 29 unique studies with 2074 unique patients were eligible for the systematic review (Figure 1)^12,13,14,15,16,17,18,19,20,21,22,23,24,25,26,27,28,29,30,31,32,33,34,35,36,37,38,39,40^; 13 studies with 984 patients reported STFS at 1 or 2 years and were included in the meta-analysis.^13,14,15,18,19,20,21,22,24,30,33,39,40^ Eight studies (29%) were prospective,^12,14,15,18,19,20,26,37^ of which 3 (11%) were randomized clinical trials.^15,18,37^ In 15 studies,^13,14,15,17,18,19,21,22,23,26,27,29,30,32,33^ all patients were treated with metastasis-directed SBRT without immediate systemic therapy, while in 13 studies,^12,16,20,24,25,28,31,35,36,37,38,39,40^ this was the case only for a defined subgroup or study arm. The sample sizes for patients treated with SBRT and no up-front systemic therapy ranged from 13 to 252. Prostate cancer was the most frequently studied tumor type (n = 14),^12,13,14,19,20,21,23,24,25,26,27,28,36,37^ followed by renal cell carcinoma (n = 2)^15,18^ and head and neck cancers (n = 2).^31,38^ Eight studies^16,17,22,32,33,34,35,40^ were tumor agnostic. Study design, patient numbers, and reported outcomes of studies included in the systematic review are shown in Table 1. The detailed inclusion and exclusion criteria are summarized in Table 2.

**Table 1.** Study Design, Sample Size, and Reported Outcomes

Source	Design	Patients without systemic therapy	Outcome
**Prostate cancer**			
See et al,^37^ 2024	Prospective single-arm phase 2 study	164 (Subgroup)	Median STFS, 67.9 mo
Mohan et al,^23^2023	Retrospective single-institutional study	103 (All)	Median STFS, 66 mo
Baron et al,^12^2023	Retrospective multi-institutional study	81 (Subgroup)	Median STFS, 29.7 mo; median PFS, 18.6 mo
Werensteijn-Honingh et al,^19^2021^a^	Retrospective single-institutional and prospective cohort study	90 (All)	Median PFS, 16 mo
Moyer et al,^24^2019^a^	Retrospective study	18 (Subgroup)	1-y STFS, 78%; 1-y PFS, 69%
Siva et al,^20^ 2018^a^	Prospective single-arm study	22 (Subgroup)	2-y STFS, 48%
Bouman-Wammes et al,^25^ 2017	Retrospective study	43 (Subgroup)	Median STFS, 17.3 mo; median PFS, 36.41 mo
Pasqualetti et al,^26^ 2016	Prospective study	29 (All)	Median STFS, 39.7 mo
Ingrosso et al,^36^ 2017	Retrospective study	21 (Subgroup)	Median STFS, 26.18 mo
Ost et al,^27^ 2016	Retrospective multi-institutional study	119 (All)	Median STFS, 28 mo; median PFS, 18 mo
Decaestecker et al,^14^ 2014^a^	Prospective study	50 (All)	Median STFS, 25 mo; 1-y STFS, 82%; median PFS, 19 mo; 1-y PFS, 64%
Mazzola et al,^21^ 2021^a^	Retrospective multi-institutional study	88 (All)	Median STFS, 22.8 mo; 1-y STFS, 89.6%; 1-y OS, 100%; median PFS, 22.8; 1-y PFS, 63%
Berkovic et al,^13^ 2013^a^	Retrospective study	24 (All)	Median STFS, 38 mo; 1-y STFS, 82%; 1-y OS, 100%; median PFS, 18 mo; 1-y PFS, 72%
Phillips et al,^28^ 2020	Randomized, 2-arm, phase 2 trial	36 (Subgroup)	Median PFS not reached
**Renal cell cancer**			
Hannan et al,^18^ 2022^a^	Prospective single-arm phase 2 study	23 (All)	1-y STFS, 91.3%; 1-y OS, 95.7%; 1-y PFS, 82.6%
Tang et al,^15^ 2021^a^	Prospective single-arm phase 2 study	30 (All)	1-y STFS, 82%; 1-y OS, 100%; median PFS, 22.7 mo; 1-y PFS, 64%
**Bladder cancer**			
Augugliaro et al,^29^ 2019	Retrospective study	13 (All)	Median PFS, 5.8 mo
**Soft tissue sarcoma**			
Loi et al,^30^ 2018^a^	Retrospective study	16 (All)	Median STFS, 28 mo; median OS, 69 mo; median PFS, 17 mo
**Head and neck cancer**			
Mohamed et al,^38^ 2024	Retrospective study	16 (Subgroup)	Median STFS, 23.9 mo
Thariat et al,^31^ 2025	Randomized multi-institutional, open-label phase 2 trial	34 (Subgroup)	Median STFS, 4.9 mo; median OS, 41.1 mo; 1-y OS, 85.3%; median PFS, 7.4 mo; 1-y PFS, 39.0%
**Gynecological cancer**			
Donovan et al,^39^ 2024^a^	Retrospective study	178 (Subgroup)	Median STFS, 21.7 mo; 1-y STFS, 66.2%
**Various primary tumors**			
Burkon et al,^32^ 2021	Retrospective study	90 (All)	Median STFS, 14 mo; median OS, 53.1 mo; median PFS, 9.4 mo
Sogono et al,^40^ 2021^a^	Retrospective study	252 (Subgroup)	Median STFS, 25.2 mo; 1-y STFS, 70%
Shahi et al,^33^ 2020^a^	Retrospective study	51 (All)	Median STFS, 15.1 mo; median OS, 42.6 mo; median PFS, 4.9 mo
Mazzola et al,^34^ 2018	Retrospective single-institutional cohort	32 (Subgroup)	Median PFS, 31 mo
Siva et al,^17^ 2023	Randomized phase 2 trial	90 (All)	Median OS, 62.4 mo; 1-y OS, 94%; median PFS, 13.2 mo; 1-y PFS, 57%
Willmann et al,^22^ 2022^a^	Retrospective single-institutional study	142 (All)	1-y STFS, 24.6%; median OS, 48.9 mo; median PFS, 6.1 mo
Camps-Malea et al,^35^ 2023	Retrospective single-institutional study	21 (Subgroup)	Median STFS, 17 mo
Baker et al,^16^ 2024	Retrospective analysis of prospective multi-institutional, single-arm, phase 2 study	198 (Subgroup)	3-y STFS, 41%; 4-y STFS, 37%; median PFS, 19 mo; median OS not reached

Abbreviations: OS, overall survival; PFS, progression-free survival; STFS, systemic therapy–free survival.

^a^ Study included in the meta-analysis.

**Table 2.** Patient Selection as Defined in the Inclusion and Exclusion Criteria^a^

Source	Inclusion criteria	Exclusion criteria
**Prostate cancer**		
See et al,^37^ 2024	≤5 Metastases (M1a or M1b and/or N1); men aged ≤80 y; ADT-naive; staging with MRI and PET	Prior palliative radiotherapy; active local tumor in prostate bed on clinical examination or imaging
Mohan et al,^23^ 2023	≤3 Metastases; Ga68 PSMA PET detected	Less than 6 mo from definitive treatment; serum testosterone level <50 ng/mL; neoadjuvant or concurrent ADT within 6 mo of SBRT
Baron et al,^12^2023	≤5 Metastases; hormone-sensitive prostate cancer	NA
Werensteijn-Honingh et al,^19^ 2021^b^	≤5 Metastases; Ga68 PSMA-PET or CT detected; >3 mo follow up	Simultaneous local tumor recurrence; nonnodal metastases; previous polymetastatic disease; ADT to 1 y before the current diagnosis
Moyer et al,^24^ 2019^b^	≤5 Metastases; biopsy-proven prostate cancer; follow-up with PSA testing	NA
Siva et al,^20^ 2018^b^	≤3 Metastases (bone or lymph nodes); detected on bone scan or CT; ECOG performance status 0-2; prostate cancer previously radically treated	Previous high-dose radiotherapy to the proposed targets; visceral metastases; prior cytotoxic chemotherapy; any change in hormonal therapy regimen within 6 wk of registration; unstable lesions
Bouman-Wammes et al,^25^ 2017	≤5 Metastases; histologically proven prostate cancer; initially treated with curative intent; biochemical PSA relapse; hormone-sensitive prostate cancer	ADT or chemotherapy before SBRT; ≥1 other type of cancer apart from prostate cancer
Pasqualetti et al,^26^ 2016	≤3 Synchronous metastases; in F18 FMCH PET-CT	NA
Ingrosso et al,^36^ 2017	Isolated lymph node metastases of recurrent prostate cancer	NA
Ost et al,^27^ 2016	≤3 Metastases (N1 or M1a, M1b, or M1c); histologically proven prostate cancer; biochemical relapse of prostate cancer following radical local prostate treatment	Serum testosterone level <50 ng/mL; ADT for >12 mo at time of SBRT; had a biochemical relapse during active treatment with ADT; previous treatment with cytotoxic agent for prostate cancer
Decaestecker et al,^14^ 2014^b^	≤3 Metastases; histologically proven diagnosis of prostate cancer; biochemical relapse following local radical prostate cancer treatment	Serum testosterone level <50 ng/mL at time of metastases detection; neoadjuvant or concurrent ADT >1 mo with SBRT; PSA level rise while on active treatment with a luteinizing hormone releasing hormone
Mazzola et al,^21^ 2021^b^	≤3 Oligorecurrent lesions detected by F18-choline or Ga68-PSMA PET-CT; histologically proven prostate cancer; biochemical relapse after primary tumor treatment	NA
Berkovic et al,^13^ 2013^b^	≤3 Synchronous asymptomatic metastases; non–castrate-resistant metastatic prostate cancer without previous palliative systemic therapy or radiotherapy	NA
Phillips et al,^28^ 2020	≤3 Asymptomatic metastases (arisen within prior 6 mo); metastases no larger than 5.0 cm; PSA levels ≥0.5 ng/mL; testosterone levels ≥125 ng/dL; PSA level doubling time <15 mo; leukocyte level, >2000/µL; absolute neutrophil count >1000/µL; platelets >50 000/µL; 18 y of age; ECOG performance status ≤2	More than 3 y of ADT; ADT in prior 6 mo; no investigational agents during participation; PSMA-targeted PET-MRI or PET-CT in prior 6 mo if they possessed radiotracer-avid lesions; spinal cord compression; serum creatinine level >3-fold of normal upper limit; inability to lay flat during SBRT
**Renal cell cancer**		
Hannan et al,^18^ 2022^b^	≤3 Extracranial metastases; >18 y of age; pathologically proven renal cell cancer; favorable or intermediate IMDC risk group; systemic therapy-naive	NA
Tang et al,^15^ 2021^b^	≤5 Metastases; >18 y of age; ECOG performance status 0-2; ≤1 previous systemic therapy (therapy was stopped at least 1 mo before SBRT); without limitations on renal cell cancer histology	Pregnancy; comorbidities that exclude safe radiotherapy; known psychiatric or substance use disorders; presence of diffuse metastatic processes
**Bladder cancer**		
Augugliaro et al,^29^ 2019	≤5 Metastases; histological diagnosis of urothelial bladder cancer; stage 4 disease at time of radiotherapy; prior urothelial bladder cancer therapy permitted; KPS >80; life expectancy >3 mo; no concomitant systemic therapy	NA
**Soft tissue sarcoma**		
Loi et al,^30^ 2018^b^	≤3 Metastases; pathological confirmation with biopsy; no concurrent or post-SBRT chemotherapy	NA
**Head and neck cancer**		
Mohamed et al,^38^ 2024	≤5 Metastases or locoregional recurrence; de novo synchronous oligometastatic patients	Intentionally untreated primary tumor; palliative radiotherapy for symptom relief; lacking histological confirmation
Thariat et al,^31^ 2025	≤3 Metastases; de novo oligometastasis or previously treated with ablative therapy and ≥1 is present at inclusion; histologically proven controlled head and neck squamous cell carcinoma primary; ECOG performance status 0-2; life expectancy greater than 6 mo; eligible for systemic therapy with the EXTREME regimen, >18 y of age	Prior systemic therapy for metastatic disease (excluding chemotherapy for localized disease); contraindications to chemotherapy or inability to undergo effective SBRT for the metastases
**Gynecological cancer**		
Donovan et al,^39^ 2024^b^	≤5 Metastases; recurrent or metastatic tumors; oligometastatic or oligoprogressive where the remainder of metastatic lesions remained controlled; dominant lesion in eloquent area (eg, spine)	Intracranial metastases at any time prior to SBRT; primary tumor recurrences
**Various primary tumors**		
Burkon et al,^32^ 2021	≤4 Metastases; >18 y of age; KPS >70%	NA
Sogono et al,^40^ 2021^b^	≤5 Metastases; >18 y of age	Unknown primary tumor; intracranial disease
Shahi et al,^33^ 2020^b^	≤3 Metastases in the abdominopelvic space (excluding liver); >18 y of age	NA
Mazzola et al,^34^ 2018	≤5 Lung metastases; KPS >70; controlled primary tumor; metachronous oligorecurrence; oligoprogressive lung metastases; oligopersistent after systemic therapy	NA
Siva et al,^17^ 2023	≤3 Lung metastases (from any nonhematologic tumor); located away from central structures; maximal tumor size 5 cm; >18 y of age; ECOG performance status of 0-1; primary or extrathoracic disease controlled with local therapy; no systemic therapy within 3 wk of treatment or after until progression	NA
Willmann et al,^22^ 2022^b^	≤5 Extracranial metastases; >18 y of age; oligorecurrent disease; no systemic therapy 1 month before diagnosis of oligometastasis	NA
Camps-Malea et al,^35^ 2023	Oligometastatic mediastinal lymph nodes	Treatment for symptomatic purposes; patients with polymetastatic disease benefiting from a compassionate treatment
Baker et al,^16^ 2024	≤5 Oligometastases	Oligoprogressive lesions

Abbreviations: ADT, androgen deprivation therapy; BED, biologically effective dose; ECOG, Eastern Cooperative Oncology Group; EXTREME regimen, chemotherapy protocol including cetuximab, cisplatin or carboplatin, and fluorouracil; F18 FMCH PET-CT, fluorine-18 fluoromethylcholine positron emission tomography (PET)–computed tomography (CT); Ga68-PSMA PET-CT, gallium 68 prostate-specific membrane antigen PET-CT; IMDC, International Metastatic RCC Database Consortium; KPS, Karnofsky performance status; MRI, magnetic resonance imaging; NA, not applicable or not available; PSA, prostate-specific antigen; RCC, renal cell carcinoma; SBRT, stereotactic body radiotherapy.

SI conversion factors: To convert PSA to µg/L, multiply by 1.0; testosterone to nmol/L, multiply by 0.0347; leukocytes and neutrophils to 10^9^/L, multiply by 0.001.

^a^ Inclusion and exclusion criteria refer to patients treated without systemic therapy after SBRT, which may differ from the overall eligibility criteria of studies in which only a subset of patients received this treatment approach.

^b^ Included in the meta-analysis.

### Criteria for Starting Systemic Therapy

Criteria for initiating systemic therapy after SBRT were inconsistently reported and commonly left to physician discretion. Prespecified criteria were mentioned in 6 studies (21%).^12,13,14,15,16,17^ In prostate cancer studies, Baron et al^12^ left the decisions at the treating physician discretion. More stringent conditions were given by Berkovic et al^13^ who initiated ADT in case of widespread failure or a prostate-specific antigen (PSA) level rise to greater than 50 ng/mL (to convert to µg/L, multiply by 1.0), and by Decaestecker et al,^14^ who initiated therapy if more than 3 metastases were detected during follow-up even when patients were still asymptomatic.

Tang et al^15^ left the decision at the discretion of the treating physician in patients with renal cell carcinoma, but recommended starting systemic therapy at progression with 3 or more metastases, significant radiotherapy adverse effects, or the risk thereof. The tumor-agnostic studies by Baker et al^16^ and Siva et al^17^ tied initiation of systemic therapy to disease progression or patient preference, with the latter study also involving a tumor board.

### STFS

**Figure 2.**
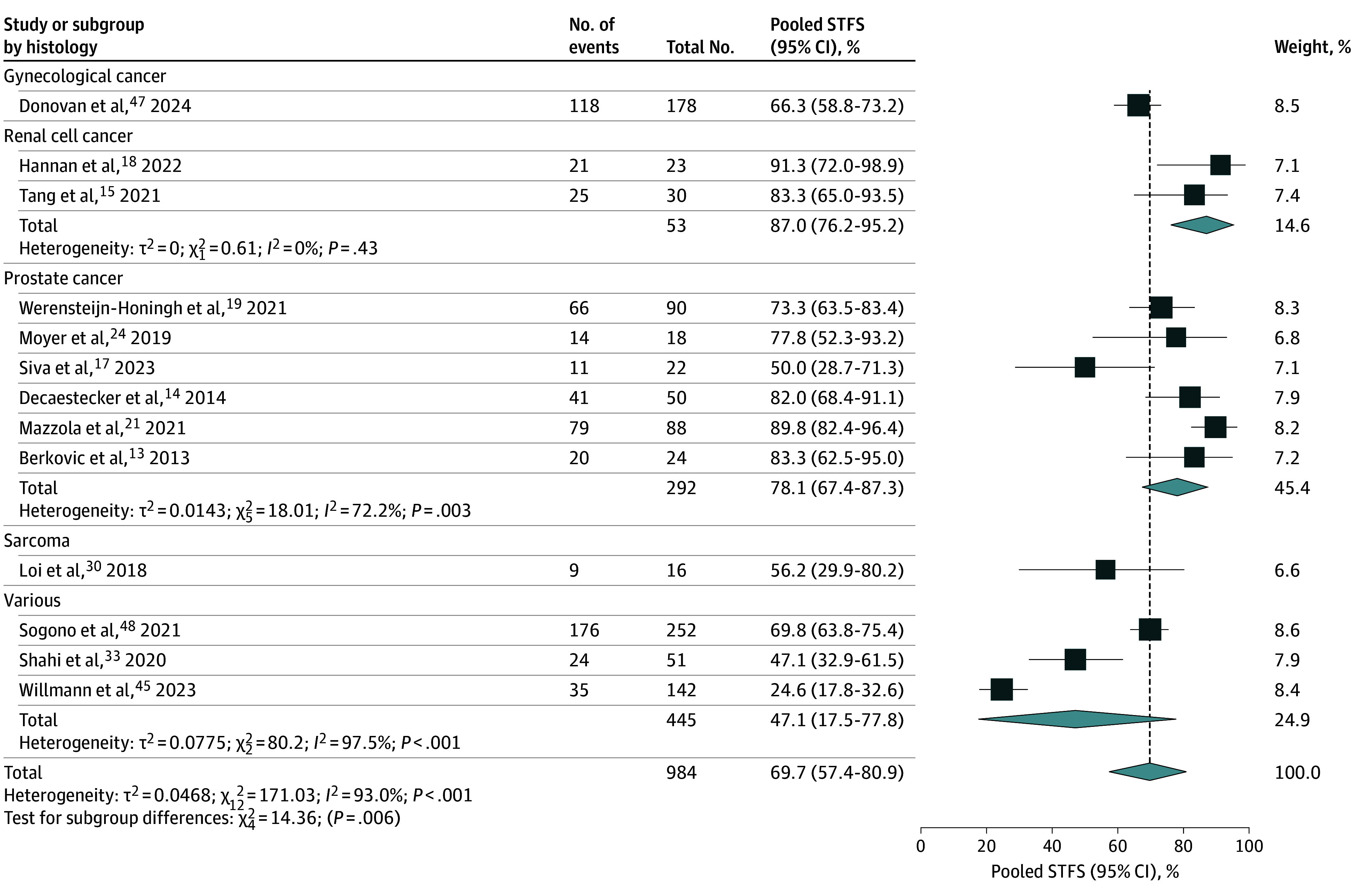
Pooled Systemic Therapy–Free Survival (STFS) at 1 or 2 Years After Stereotactic Body Radiotherapy for Oligometastatic Disease as Reported in 13 Studies

Thirteen studies^13,14,15,17,18,19,21,24,30,33,39,40,41^ (984 patients and 639 events) provided 1- or 2-year STFS data for meta-analysis (Figure 2). The pooled 1- or 2-year STFS rate was 69.7% (95% CI, 57.4%-80.9%) with considerable heterogeneity (*I*^2^ = 93.0%; *P* < .001). Subgroup analysis showed the highest STFS in renal cell cancer at 87.0% (95% CI, 76.2%-95.2%; *I*^2^ = 0%; *P* = .43), followed by prostate cancer at 78.1% (95% CI, 67.4%-87.3%; *I*^2^ = 72.2%; *P* = .003). Other cancer types demonstrated lower pooled STFS, including 66.3% (95% CI, 58.8%-73.2%) in gynecological cancer, 56.2% (95% CI, 29.9%-80.2%) in sarcoma, and 47.1% (95% CI, 17.5%-77.8%) in studies including different tumor types, the latter with considerable heterogeneity (*I*^2^ = 97.5%; *P* < .001). Meta-regression indicated that histology explained 35.7% of between-study heterogeneity (*R*^2^ = 35.7%), with considerable residual heterogeneity remaining (*I*^2^ = 86.9%; *P* < .001). While histology significantly contributed to the model overall (test of moderators: *Q*_4_ = 9.52; *P* = .049), no individual subtype differed significantly from the reference category.

**Figure 3.**
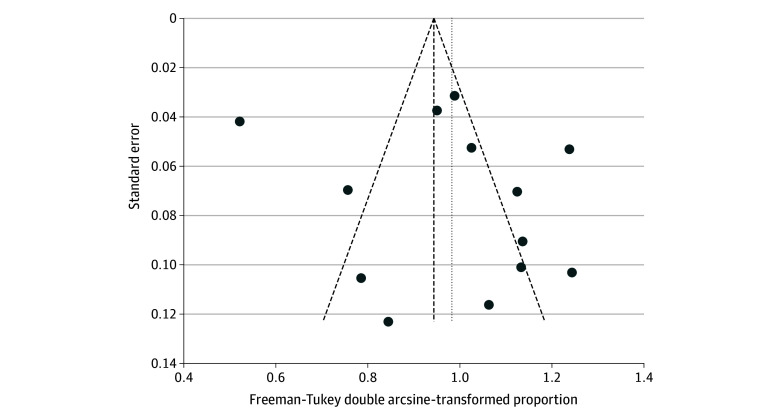
Funnel Plots of Meta-Analyses for Systemic Therapy–Free Survival at 1 or 2 Years After Stereotactic Body Radiotherapy for Oligometastatic Disease as Reported in 13 Studies

The funnel plot for STFS showed no asymmetry by visual inspection or Egger test, suggesting no publication bias (Figure 3). The risk of bias assessment using the Newcastle-Ottawa Scale demonstrated that most studies were of moderate to high quality, 12 studies as high quality (scores 7-9),^12,16,17,24,25,28,31,35,36,37,38,40^ 16 as moderate quality (scores 4-6),^13,14,15,18,19,20,21,22,23,27,29,30,32,33,34,39^ and 1 as low quality (scores ≤3),^26^ reflecting variability in study design, control of confounding, and outcome assessment (eTable 1 in Supplement 1).

To assess the consistency of findings across study designs, we performed a sensitivity analysis restricted to prospective studies (5 studies with 215 patients).^14,15,18,19,20^ Among these, the pooled 1- or 2-year STFS rate was 87.0% (95% CI, 76.2%-95.2%) for renal cell cancer and 71.1% (95% CI, 55.6%-84.5%) for prostate cancer (eFigure in Supplement 1). Heterogeneity was not important among renal cell cancer studies (*I*^2^ = 0%) but remained considerable within the prostate cancer subgroup (*I*^2^ = 72.1%). The Egger test suggested a low risk of publication bias (intercept, 1.04; 95% CI, 0.25-1.83; *t*_3_ = 0.09; *P* = .94).

To further assess the robustness of our findings, we performed a leave-one-out sensitivity analysis (eTable 2 in Supplement 1). The analysis showed minimal variation in the pooled STFS estimate when individual studies were removed. The proportion estimates ranged from 72.3% (95% CI, 69.8%-74.6%) when excluding Mazzola et al^21^ to 73.6% (95% CI, 71.8%-75.3%) when excluding Willmann et al,^22^ representing a narrow range of only 1.3 percentage points. This stability indicates that no single study disproportionately influenced the overall pooled estimate. Heterogeneity remained considerable across all leave-one-out iterations (*I*^2^ range, 83.1%-93.0%). The between-study variance (τ^2^) showed more variability, ranging from 0.0191 to 0.0453.

### Adverse Effects and Quality of Life

Across 20 studies (69%) reporting adverse events, SBRT without up-front systemic therapy was well tolerated.^13,14,15,16,18,19,21,23,24,25,26,27,28,29,30,31,32,33,34,35^ Reported rates of adverse effects of grade 3 or greater were absent in 15 of 19 studies (79%) studies and ranged from 2 of 103 (1.9%) to 3 of 34 patients (8.8%) among those that observed severe adverse events. Six (21%) studies assessed quality of life.^18,19,20,26,28,31^ Overall, SBRT alone was associated with preserved QOL across disease sites. Further details on adverse effects and QOL are provided in eTable 3 in Supplement 1.

### Repeat SBRT

Overall, 14 studies (52%)^13,14,16,18,19,21,22,23,25,26,27,30,33,36^ reported on the use of repeat SBRT to manage disease progression while deferring systemic therapy. In prostate cancer studies, repeat SBRT was reported in 16% to 42% of patients.^13,14,19,21,23,25,26,27,36^ Among patients with renal cell cancer in the study by Hannan et al,^18^ 44% received a second course of SBRT. In the sarcoma study by Loi et al,^30^ 25% of patients underwent repeat SBRT. Among studies including various primary tumors, the proportion of patients receiving repeat SBRT ranged from 18% to 34%.^16,22,33^

### Factors Associated With STFS

Factors associated with STFS were reported in 7 studies (24%),^12,13,14,19,22,30,32^ mostly focusing on prostate cancer. Several studies found that lower PSA levels, favorable response after SBRT, and shorter doubling time for PSA levels were associated with longer STFS.^12,14,19^ One study^13^ also linked a lower Gleason score to prolonged ADT-free survival. Given the heterogeneity across tumor types, these findings are hypothesis generating and primarily applicable to prostate cancer.

### Freedom From Metastases

The proportion of patients remaining metastasis-free at last follow-up was reported in 7 studies (24%).^23,24,29,30,32,36,37^ For prostate cancer, freedom from metastases ranged from 23% (median follow-up, 68 months) to 76% (median follow-up, 30 months).^23,24,36,37^ Lower rates of freedom from metastases were reported for soft tissue sarcoma (6%; median follow-up, 36 [IQR, 18-71] months^30^) and bladder cancer (23%; median follow-up, 25 [range, 3-43] months^29^). In the study by Burkon et al^32^ encompassing multiple cancer types, the proportion of patients remaining free from metastases was 38% (median follow-up, 35 months).

## Discussion

To our knowledge, this is the first systematic review and meta-analysis of metastasis-directed SBRT without up-front systemic therapy in oligometastatic cancer. Our findings suggest that SBRT alone might be associated with substantial STFS, particularly in prostate and renal cell cancers, with a favorable safety profile.

With a pooled 1- or 2-year STFS rate of 69.7% across various cancer types, our study suggests that SBRT alone may be an acceptable option instead of systemic therapy. Notably, patients with renal cell carcinoma exhibited the highest STFS rate at 87.0%, followed by those with prostate cancer at 78.1%. However, in the absence of randomized clinical trials for renal cell cancer comparing SBRT alone with observation, the extent to which systemic therapy deferral is influenced by SBRT vs indolent tumor biology remains unclear. Results of the 2 prospective single-institution studies by Tang et al^15^ and Hannan et al^18^ with small sample sizes (30 and 23 patients, respectively) should be confirmed by randomized clinical trials, involving multiple centers or trial cooperative groups to facilitate enrollment of patients with the relatively rare disease. For prostate cancer, however, the use of metastasis-directed therapy alone is supported by randomized clinical trial data. The phase 2 randomized clinical Observation Versus Stereotactic Ablative Radiation for Oligometastatic Prostate Cancer (ORIOLE) trial by Phillips et al,^28^ which compared SBRT alone with observation in patients with castration-sensitive metachronous oligometastatic prostate cancer, did not report STFS and was therefore included in the systematic review but not in the meta-analysis. Median PFS with SBRT was not reached compared with 5.8 months with observation (hazard ratio [HR], 0.30; 95% CI, 0.11-0.81; *P* = .002). Similarly, the phase 2 randomized clinical Salvage Treatment or Active Clinical Surveillance for Oligometastatic Prostate Cancer (STOMP) trial,^42^ although not included in our meta-analysis due to the use of both SBRT and surgical resection without stratified reporting, is a pivotal study supporting the role of local therapy alone. In STOMP, metastasis-directed therapy significantly prolonged ADT-free survival compared with surveillance in patients with metachronous oligometastatic prostate cancer (median, 21 [80% CI, IQR, 14-29] vs 13 [80% CI, 12-17] months; HR, 0.60 [80% CI, 0.40-0.90]; log rank *P* = .11). A pooled analysis of the STOMP and ORIOLE trials with long-term follow-up confirmed the PFS benefit with metastasis-directed local therapy compared with observation (HR, 0.44; [95% CI, 0.29-0.66]; *P* < .001).^43^ The authors also identified high-risk genomic alterations (pathogenic somatic mutations in *ATM*, *BRCA1/2*, *Rb1*, and *TP53*) that were associated with a greater PFS benefit from metastasis-directed therapy. Randomized clinical trials and biomarker studies are needed across oligometastatic tumor types to assess the value of SBRT without up-front systemic therapy as a function of genomic profiles.

Metastasis-directed SBRT without up-front systemic therapy was generally well tolerated across studies included in our systematic review. Across the included studies reporting adverse events (20 [69%]), grade 3 or higher toxic effects were absent in most studies among the 20 that reported adverse events (78.9%), which reported in 1.9% to 8.8% of patients. This favorable adverse effect profile supports the use of SBRT as a viable option for patients who may not tolerate systemic therapies due to comorbidities or personal preferences. The tolerability of metastasis-directed SBRT is well documented, with a low incidence of severe toxic effects in a systematic review of prospective studies on oligometastatic disease.^44^ However, a potential risk of severe adverse events must be carefully considered, with 4.5% lethal adverse events observed in the randomized Stereotactic Ablative Radiotherapy for Comprehensive Treatment of Oligometastatic Tumors (SABR-COMET) trial.^45^ In clinical practice, however, rates are likely much lower, such as less than 0.5% reported in the population-based SABR-5 trial.^46^ Of note, these trials included patients receiving metastasis-directed SBRT and systemic therapy (although systemic treatments were stopped for 3 to 4 weeks around SBRT); thus, rates of adverse events with SBRT alone might be even lower.

Our findings suggest that—although reported in only 5 studies (17%)— QOL is preserved or minimally impacted by SBRT alone. In the randomized trial by Phillips et al,^28^ patients with prostate cancer treated with SBRT demonstrated stable pain scores and functional QOL domains during 6 months, compared with patients under observation. These findings are consistent with the prospective observational OligoCare cohort study,^47^ which reported that metastasis-directed SBRT had no detrimental impact on QOL in patients with oligometastatic prostate cancer, half of whom did not receive concomitant hormonal therapy. On the other hand, in the randomized clinical trial by Thariat et al,^31^ patients receiving SBRT alone for head and neck cancer oligometastases experienced significantly less severe QOL deterioration compared with those receiving combined chemotherapy and SBRT. Notably, chemotherapy is no longer considered the standard of care for these patients, as immune checkpoint inhibitors are now the first-line treatment for metastatic head and neck cancers.^48^ As the adverse effect profile and thus likely QOL impact of immune checkpoint inhibitors differs from chemotherapy, the role of SBRT alone needs to be reconsidered in future studies. In renal cell carcinoma, the prospective single-arm study by Hannan et al^18^ reported largely stable QOL to 15 months post SBRT, reinforcing the potential of SBRT to preserve functional outcomes while delaying systemic therapy. Standardized QOL assessment in randomized clinical trials is essential to comprehensively evaluate the patient-reported benefits of SBRT in this context.

The ability of SBRT to defer systemic therapy has important clinical implications, particularly for patients with limited metastatic burden and favorable tumor biology. By postponing systemic therapy, patients may avoid or delay the associated toxic effects and preserve their quality of life. Moreover, the role of repeat SBRT in managing limited progressive disease is noteworthy, delivered in 14% to 44% of patients. A dedicated retrospective study on repeat SBRT for oligometastatic disease reported low rates of adverse effects, even with multiple consecutive courses and in combination with systemic therapy.^41^ However, prospective data evaluating the safety and efficacy of repeat SBRT are lacking. Implementing predefined criteria for initiating systemic therapy is crucial to standardize treatment sequencing and optimize patient outcomes. These criteria were often undefined or left to physician discretion, underscoring the need for clear guidelines in clinical practice.

### Limitations

This study has several limitations. The included studies enrolled a heterogeneous population, including patients with de novo systemic treatment for naive oligometastatic disease as well as those who had received prior lines of systemic therapy. This variability should be considered when interpreting the generalizability of our findings. The predominance of retrospective studies, with only a few prospective or randomized clinical trials, limits control over selection bias and unmeasured confounders. Considerable heterogeneity was observed across patient populations, treatment protocols, and criteria for defining oligometastatic disease, complicating the comparability of results. Criteria for initiating systemic therapy varied widely, often being left to physician discretion without predefined guidelines. This lack of standardization in treatment sequencing introduces variability in outcomes and hinders the development of uniform clinical protocols. Staging and follow-up examinations were not predefined, leading to potential variability in disease assessment and treatment decisions.

## Conclusions

In this systemic review and meta-analysis, metastasis-directed SBRT without up-front systemic therapy was associated with clinically meaningful deferral of systemic treatment in patients with oligometastatic prostate and renal cell cancer. This strategy was associated with a low incidence of severe adverse events, potentially preserving quality of life. Randomized clinical trials are needed to confirm outcomes and refine treatment strategies. Prognostic and predictive biomarkers should be explored to guide patient selection.

## Notes

Supplement 1. **eMethods 1.** Study Design and Data Management **eMethods 2.** Interpretation of *I*^2^ Statistic for Heterogeneity Assessment, Following Cochrane Handbook Guidelines **eFigure.** Pooled Systemic Therapy–Free Survival (STFS) at 1 or 2 Years After Stereotactic Body Radiotherapy (SBRT) for Oligometastatic Disease as Reported in the Prospective Studies in the Sensitivity Analysis **eTable 1.** Newcastle-Ottawa Scale (NOS) for Assessing the Risk of Bias **eTable 2.** Leave-One-Out Sensitivity Analysis of Systemic Therapy–Free Survival (STFS) at 1 to 2 Years Following Stereotactic Body Radiotherapy (SBRT) for Oligometastatic Cancer **eTable 3.** Adverse Effects (Grade 3 or Higher) and Quality of Life (QOL)
eReferences

Supplement 2.
Data Sharing Statement
