# Supplementary material for: Stereotactic Body Radiotherapy Without Systemic Therapy for Oligometastatic Cancer: A Systematic Review and Meta-Analysis
Source: JAMA Netw Open. 2025 Dec 29;8(12):e2549685. doi: 10.1001/jamanetworkopen.2025.49685 (PMC12750244; doi:10.1001/jamanetworkopen.2025.49685)
Supplement: Supplement 1. — eMethods 1. Study Design and Data Management eMethods 2. Interpretation of I2 Statistic for Heterogeneity Assessment, Following Cochrane Handbook Guidelines eFigure. Pooled Systemic Therapy–Free Survival (STFS) at 1 or 2 Years After Stereotactic Body Radiotherapy (SBRT) for Oligometastatic Disease as Reported in the Prospective Studies in the Sensitivity Analysis eTable 1. Newcastle-Ottawa Scale (NOS) for Assessing the Risk of Bias eTable 2. Leave-One-Out Sensitivity Analysis of Systemic Therapy–Free Survival (STFS) at 1 to 2 Years Following Stereotactic Body Radiotherapy (SBRT) for Oligometastatic Cancer eTable 3. Adverse Effects (Grade 3 or Higher) and Quality of Life (QOL) eReferences [file jamanetwopen-e2549685-s001.pdf]

Supplementary Online Content

Willmann J, von Wachter C, Zehnder R, et al. Stereotactic body radiotherapy without systemic therapy for oligometastatic cancer: a systematic review and meta-analysis. *JAMA Netw Open.* 2025;8(12):e2549685. doi:10.1001/jamanetworkopen.2025.49685

eMethods 1. Study Design and Data Management
eMethods 2. Interpretation of I^2 Statistic for Heterogeneity Assessment, Following Cochrane Handbook Guidelines
eFigure. Pooled Systemic Therapy–Free Survival (STFS) at 1 or 2 Years After Stereotactic Body Radiotherapy (SBRT) for Oligometastatic Disease as Reported in the Prospective Studies in the Sensitivity Analysis
eTable 1. Newcastle-Ottawa Scale (NOS) for Assessing the Risk of Bias
eTable 2. Leave-One-Out Sensitivity Analysis of Systemic Therapy–Free Survival (STFS) at 1 to 2 Years Following Stereotactic Body Radiotherapy (SBRT) for Oligometastatic Cancer
eTable 3. Adverse Effects (Grade 3 or Higher) and Quality of Life (QOL)
eReferences

This supplementary material has been provided by the authors to give readers additional information about their work.

© 2025 Willmann J et al. *JAMA Network Open*.

eMethods 1. Study Design and Data Management

Study design

The Population, Intervention, Control, Outcomes and Study Design method was used to define literature inclusion criteria[1]. The Preferred Reporting Items for Systematic Reviews and Meta-analyses (PRISMA) reporting guideline was used[2]. In addition, the Meta-analysis of Observational Studies in Epidemiology (MOOSE) reporting guideline were used[3].

Population, Intervention, Control, Outcome, Study Design (PICOS) Inclusion Criteria.

**Population**	Patients with oligometastatic cancer
**Interventions**	SBRT to all metastases as treatment
**Control**	Either no control group (i.e. single-arm study); or a multi-arm study that compares SBRT alone to other combination or single modality treatments (e.g. systemic therapy alone, or SBRT and systemic therapy in combination)
**Outcomes**	Systemic therapy-free survival, median and 1-year and 2-years rate<br><br>Progression-free survival, median and 1-year and 2-years rate<br><br>Overall survival, median and 1-year and 2-years rate
**Study design**	Retrospective studies with ≥10 participants, all prospective studies of any phase published after 01/2009

Eligibility Criteria

Inclusion Criteria

- Prospective studies of any sample size (multi-arm or single-arm) or retrospective studies with at least 10 participants
- Patients with oligometastatic cancer, irrespective of primary tumor type
- All patients in a defined subgroup or treatment arm underwent SBRT to all metastases without immediate systemic therapy
- At least one of the primary or secondary outcome measures reported for the group of patients treated with SBRT without systemic therapy
    - For inclusion in the meta-analysis: Reporting systemic therapy-free survival at 1 or 2 years

Exclusion Criteria

- Non-original research (reviews, editorials), unfinished studies published as abstracts
- Studies including metastasis-directed treatment other than SBRT
- Studies involving non-human subjects
- Works not published in English

Databases

PubMed, EMBASE

Search Strategy

A comprehensive search strategy the following keywords were used:
(oligometastasis OR oligometastatic OR oligoprogression OR oligoprogressive OR oligorecurrent OR oligopersistent) AND (stereotactic AND radiotherapy)

The strategy has been peer-reviewed, using the Peer Review of Electronic Search Strategies (PRESS) guideline.[4]

We conducted both forward and backward citation searches by reviewing reference lists of included studies, and identifying studies that cited the included studies via citation indices.

Data Management

Selection Process

The selection of studies (screening, eligibility, and inclusion) were performed by two independent reviewers (CvW and RZ), and conflicts discussed with another (JW).

Data Collection Process

The online plattform rayyan.ai was used for screening and data extraction, including study characteristics, participant details, interventions, and outcomes.

© 2025 Willmann J et al. *JAMA Network Open*.

eMethods 2. Interpretation of I^2 Statistic for Heterogeneity Assessment, Following Cochrane Handbook Guidelines

- 0% to 40%: might not be important
- 30% to 60%: may represent moderate heterogeneity
- 50% to 90%: may represent substantial heterogeneity
- 75% to 100%: considerable heterogeneity

These categories are not absolute and must be interpreted in the context of study characteristics and the direction of effects.

© 2025 Willmann J et al. *JAMA Network Open*.

eFigure. Pooled Systemic Therapy–Free Survival (STFS) at 1 or 2 Years After Stereotactic Body Radiotherapy (SBRT) for Oligometastatic Disease as Reported in the Prospective Studies in the Sensitivity Analysis

Study or Subgroup	Events	Total	Weight	IV, Random, 95% CI
**Histology = Renal cell**				
Hannan	21	23	16.8%	0.913 [0.720; 0.989]
Tang	25	30	18.8%	0.833 [0.650; 0.935]
**Total (95% CI)**		**53**	**35.7%**	**0.870 [0.762; 0.952]**
Heterogeneity: $Tau^2 = 0$; $Chi^2 = 0.61$, df = 1 (P = 0.4343); $I^2 = 0\%$				
**Histology = Prostate**				
Werensteijn-Honingh	66	90	25.5%	0.733 [0.634; 0.831]
Siva	11	22	16.5%	0.500 [0.287; 0.713]
Decaestecker	41	50	22.3%	0.820 [0.688; 0.919]
**Total (95% CI)**		**162**	**64.3%**	**0.711 [0.556; 0.845]**
Heterogeneity: $Tau^2 = 0.0136$; $Chi^2 = 7.17$, df = 2 (P = 0.0277); $I^2 = 72.1\%$				
**Total (95% CI)**		**215**	**100.0%**	**0.774 [0.660; 0.871]**
Heterogeneity: $Tau^2 = 0.0125$; $Chi^2 = 11.94$, df = 4 (P = 0.0178); $I^2 = 66.5\%$				
Test for subgroup differences: $Chi^2 = 3.12$, df = 1 (P = 0.0775)				

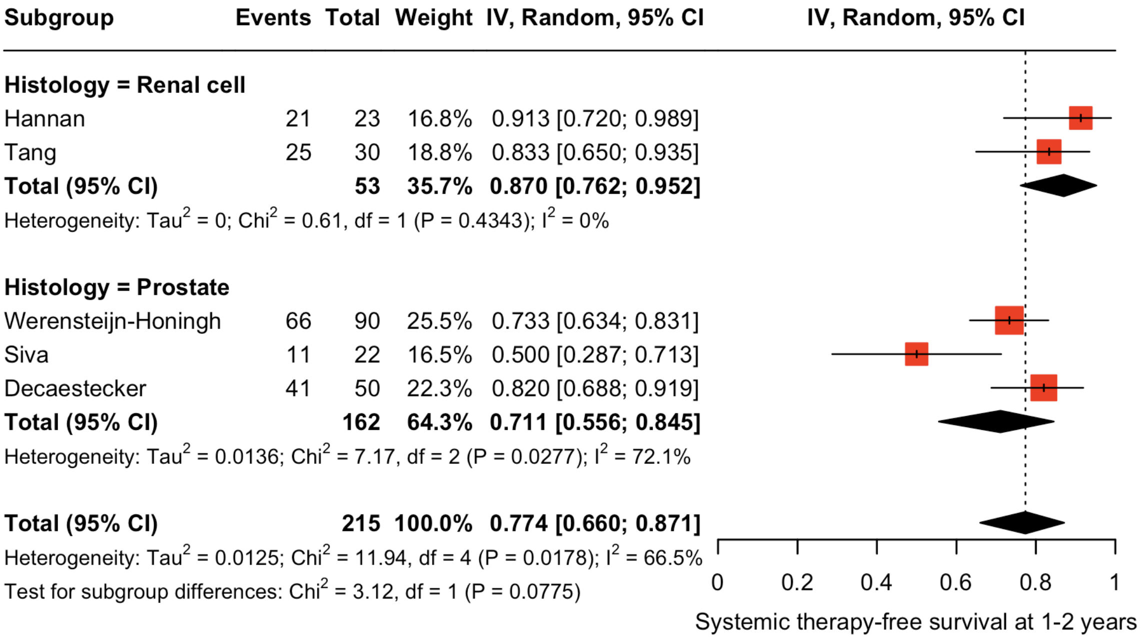

© 2025 Willmann J et al. *JAMA Network Open*.

eTable 1. Newcastle-Ottawa Scale (NOS) for Assessing the Risk of Bias

NOS scores reflect risk of bias: high quality (7–9), moderate quality (4–6), low quality (≤3). Studies included in the meta-analysis are marked with an asterisk (*).

First author (year)	Selection	Comparability	Outcomes	Total
**Prostate cancer**				
See (2024)[5]	4	1	3	8
Mohan (2023)[6]	2	0	2	4
Baron (2023)[7]	4	1	2	7
Werensteijn-Honingh (2021)[8] *	3	0	2	5
Moyer (2019)[9] *	4	1	2	7
Siva (2018) [10] *	4	1	3	8
Bouman-Wammes (2017)[11]	4	1	3	8
Pasqualetti (2016)[12]	2	0	1	3
Ingrosso (2016)[13]	3	1	3	7
Ost (2015)[14]	3	0	3	6
Decaestecker (2014)[15] *	2	0	2	4
Mazzola (2021)[16] *	2	0	2	4
Berkovic (2013) [17] *	2	0	3	5
Phillips (2020) [18]	4	1	2	7
**Renal cell cancer**				
Hannan (2022)[19] *	3	0	3	6
Tang (2021)[20] *	3	0	2	5
**Bladder cancer**				
Augugliaro (2019)[20,21]	2	0	3	5
**Soft tissue sarcoma**				
Loi (2018)[22] *	3	0	3	6
**Head and neck cancer**				
Mohamed (2023)[23]	4	1	3	8
Thariat (2025)[24]	4	1	3	8
**Gynecological malignancies**				
Donovan (2024)[25] *	4	0	2	6

© 2025 Willmann J et al. *JAMA Network Open*.

**Various primary tumors**				
Burkon (2021)[26]	3	0	2	5
Sogono (2020)[27] *	4	1	3	8
Shahi (2020)[28] *	3	0	3	6
Mazzola (2018)[29]	3	1	2	6
Siva (2023)[29,30]	3	0	3	6
Willmann (2022)[31] *	3	0	3	6
Camps-Malea (2022)[32]	4	1	3	8
Baker (2024)[33]	4	1	3	8

© 2025 Willmann J et al. *JAMA Network Open*.

eTable 2. Leave-One-Out Sensitivity Analysis of Systemic Therapy–Free Survival (STFS) at 1 to 2 Years Following Stereotactic Body Radiotherapy (SBRT) for Oligometastatic Cancer

Study Removed	Primary Tumor	Sample Size	STFS Events	STFS Proportion (95% CI)	I^2 (%)	τ^2	P value
**Donovan**[25]	Gynecological	178	118	72.8% (70.2-75.3%)	91.8	0.0451	<0.001
**Hannan**[19]	Renal cell	23	21	72.4% (69.9-74.7%)	92.1	0.0392	<0.001
**Werensteijn-Honingh**[8]	Prostate	90	66	72.7% (70.1-75.2%)	92.5	0.0449	<0.001
**Moyer**[8]	Prostate	18	14	72.6% (70.1-75.1%)	93.0	0.0438	<0.001
**Siva**[10]	Prostate	22	11	73.1% (70.6-75.4%)	92.6	0.0417	<0.001
**Decaestecker**[15]	Prostate	50	41	72.5% (69.9-74.9%)	92.5	0.0428	<0.001
**Mazzola**[16]	Prostate	88	79	72.3% (69.8-74.6%)	91.2	0.0378	<0.001
**Berkovic**[17]	Prostate	24	20	72.5% (70.0-74.9%)	92.7	0.0427	<0.001
**Tang**[20]	Renal cell	30	25	72.5% (70.0-74.9%)	92.7	0.0426	<0.001
**Loi**[22]	Sarcoma	16	9	73.0% (70.4-75.3%)	92.9	0.0431	<0.001
**Sogono**[27]	Various	252	176	72.8% (70.1-75.2%)	91.3	0.0453	<0.001
**Shahi**[28]	Various	51	24	73.1% (70.7-75.5%)	92.0	0.0401	<0.001
**Willmann**[31]	Various	142	35	73.6% (71.8-75.3%)	83.1	0.0191	<0.001

© 2025 Willmann J et al. *JAMA Network Open*.

eTable 3. Adverse Effects (Grade 3 or Higher) and Quality of Life (QOL)

NA: not applicable, as results for not reported. Studies included in the meta-analysis are marked with an asterisk (*). Abbreviations: NA: not applicable, as study did not report adverse effects or QoL.

**First author (year)**	**Adverse effects (grade 3 or higher)**	**Quality of life**
**Prostate cancer**		
See (2024)[5]	NA	NA
Mohan (2023)[6]	2/103 grade 3 (rib fracture and lymphoedema)	NA
Baron (2023)[7]	NA	NA
Werensteijn-Honingh (2021)[8] *	none	- Grade 1 fatigue was most predominant acute adverse effects (34%)<br>- QoL assessed using EORTC C30, EuroQol EQ-5D-5L and Multidimensional Fatigue Inventory (MFI)<br>- QoL analysis showed mild, transient increase in fatigue at 1-4 weeks after SBRT<br>- Overall health status and physical functioning were unaffected<br>- Fatigue subdomains showed mainly reduced activity after SBRT, resolved at 6 months
Moyer (2019)[9] *	none	NA
Siva (2018) [10] *	NA	- QoL assessed using EORTC QLQ-C30 and QLQ-BM22 at 1, 3, 12, and 24 months<br>- No significant difference from baseline QoL observed overall<br>- Only pain subscales at 24 months and functional interference showed minimal important difference from baseline<br>- QoL measures were maintained with single fraction SABR treatment strategy

© 2025 Willmann J et al. *JAMA Network Open*.

		- Treatment-related adverse events in 67% of patients, mostly grade 1-2<br>- Pain characteristics and painful sites increased only at 24-month timepoint<br>- Overall health status remained stable throughout follow-up
Bouman-Wammes (2017)[11]	none	NA
Pasqualetti (2016)[12]	none	- QoL assessed using EORTC QLQ-C30 questionnaire<br>- Geriatric evaluation did not show any worsening during the course of the study<br>- No treatment-related deterioration in QoL measures
Ingrosso (2016)[13]	NA	NA
Ost (2016)[14]	none	NA
Decaestecker (2014)[15] *	none	NA
Mazzola (2021)[16] *	none	NA
Berkovic (2013)[17] *	none	NA
Phillips (2020)[18]	none	- QoL assessed using Brief Pain Inventory (Short Form)<br>- No differences in Brief Pain Inventory scores were observed between arms or within either arm across time<br>- SABR did not appear to affect quality of life measures<br>- QoL maintained throughout the study period in both treatment arms<br>- No significant changes in pain scores or functional status reported
**Renal cell cancer**		
Hannan (2022)[19] *	none	- EuroQol Group's five-level measure (EQ-5D-5L), Functional Assessment of Cancer Therapy – General (FACT-G), and Functional Assessment of

© 2025 Willmann J et al. *JAMA Network Open*.

		Cancer Therapy – Kidney Symptom Index (FKSI)<br>- QoL was largely preserved throughout treatment - most questionnaire responses showed no significant change from baseline at 3, 9, and 15-month follow-up<br>- Only two statistically significant declines observed: FKSI at 3 months ($p = 0.035$) - but recovered at 9 and 15 months, and FACT-G total score at 15 months ($p = 0.042$)
Tang (2021)[20] *	1/30 grade 4 (hyperglycaemia) and 2/30 grade 3 (back pain and muscle weakness)	NA
**Bladder cancer**		
Augugliaro (2019)[20,21]	none	NA
**Soft tissue sarcoma**		
Loi (2018)[22] *	none	NA
**Head and neck cancer**		
Mohamed (2023)[23]	NA	NA
Thariat (2025)[24]	3/34 grade 3 and 4	- EORTC QLQ-C30 and EORTC QLQ-Head and Neck 35<br>- At one year, approximately 55% of patients in the SBRT-alone arm and 53% in the chemotherapy and SBRT arm were alive without definitive QoL deterioration<br>- Median QoL deterioration in the SBRT-alone arm was minimal (16.7; IQR: 16.7–41.7) compared to the chemotherapy and SBRT-arm (50.0; IQR: 41.7–66.7)<br>- Physical functioning and sexuality scores were notably better in the SBRT-alone arm compared to the chemotherapy and SBRT-arm<br>- Severe (Grade 3-4) toxicities were lower with SABR alone (8.8%)

© 2025 Willmann J et al. *JAMA Network Open*.

		compared to chemotherapy and SBRT (60%). Patients experiencing severe toxicities were more likely to have deteriorated QoL outcomes
**Gynecological malignancies**		
Donovan (2024)[25] *	NA	NA
**Various primary tumors**		
Burkon (2021)[26]	none	NA
Sogono (2020)[27] *	NA	NA
Shahi (2020)[28] *	1/51 grade 3 (nausea and dehydration)	NA
Mazzola (2018)[29]	none	NA
Siva (2023)[29,30]	NA	NA
Willmann (2022)[31] *	NA	NA
Camps-Malea (2022)[32]	none	NA
Baker (2024)[33]	8/198 grade 3 (bone metastasis fracture, fatigue, pain, nausea, hydronephrosis, urinary retention, ascites)	NA

© 2025 Willmann J et al. *JAMA Network Open*.

eReferences

1. Huang X, Lin J, Demner-Fushman D. Evaluation of PICO as a knowledge representation for clinical questions. *AMIA Annu Symp Proc*. 2006;2006:359-363.

2. Moher D, Liberati A, Tetzlaff J, Altman DG, PRISMA Group. Preferred reporting items for systematic reviews and meta-analyses: the PRISMA statement. *PLoS Med*. 2009;6(7):e1000097.

3. Stroup DF, Berlin JA, Morton SC, et al. Meta-analysis of observational studies in epidemiology: a proposal for reporting. Meta-analysis Of Observational Studies in Epidemiology (MOOSE) group. *JAMA*. 2000;283(15):2008-2012.

4. McGowan J, Sampson M, Salzwedel DM, Cogo E, Foerster V, Lefebvre C. PRESS Peer Review of Electronic Search Strategies: 2015 Guideline Statement. *J Clin Epidemiol*. 2016;75:40-46.

5. See AW, Conway P, Frydenberg M, et al. Five-year outcomes of fractionated stereotactic body radiotherapy for oligometastatic prostate cancer from the TRANSFORM phase II trial. *Int J Cancer*. 2024;155(7):1248-1256.

6. Mohan R, Kneebone A, Eade T, et al. Long-term outcomes of SBRT for PSMA PET detected oligometastatic prostate cancer. *Radiat Oncol*. 2023;18(1):127.

7. Baron D, Pasquier D, Pace-Loscos T, et al. Systemic therapy escalation after stereotactic body radiation therapy for oligometastatic hormone-sensitive prostate cancer. *Clin Transl Radiat Oncol*. 2023;43:100673.

8. Werensteijn-Honingh AM, Wevers AFJ, Peters M, et al. Progression-free survival in patients with Ga-PSMA-PET-directed SBRT for lymph node oligometastases. *Acta Oncol*. 2021;60(10):1342-1351.

9. Moyer CL, Phillips R, Deek MP, et al. Stereotactic ablative radiation therapy for oligometastatic prostate cancer delays time-to-next systemic treatment. *World J Urol*. 2019;37(12):2623-2629.

10. Siva S, Bressel M, Murphy DG, et al. Stereotactic abative body radiotherapy (SABR) for oligometastatic prostate cancer: A prospective clinical trial. *Eur Urol*. 2018;74(4):455-462.

11. Bouman-Wammes EW, van Dodewaard-De Jong JM, Dahele M, et al. Benefits of Using Stereotactic Body Radiotherapy in Patients With Metachronous Oligometastases of Hormone-Sensitive Prostate Cancer Detected by [18F]fluoromethylcholine PET/CT. *Clin Genitourin Cancer*. 2017;15(5):e773-e782.

12. Pasqualetti F, Panichi M, Sainato A, et al. [(18)F]Choline PET/CT and stereotactic body radiotherapy on treatment decision making of oligometastatic prostate cancer patients: preliminary results. *Radiat Oncol*. 2016;11:9.

13. Ingrosso G, Trippa F, Maranzano E, et al. Stereotactic body radiotherapy in oligometastatic prostate cancer patients with isolated lymph nodes involvement: a two-institution experience. *World J Urol*. 2017;35(1):45-49.

14. Ost P, Jereczek-Fossa BA, Van As N, et al. Progression-free Survival Following

© 2025 Willmann J et al. *JAMA Network Open*.

Stereotactic Body Radiotherapy for Oligometastatic Prostate Cancer Treatment-naive Recurrence: A Multi-institutional Analysis. *Eur Urol*. 2016;69(1):9-12.

15. Decaestecker K, De Meerleer G, Lambert B, et al. Repeated stereotactic body radiotherapy for oligometastatic prostate cancer recurrence. *Radiat Oncol*. 2014;9(1):135.

16. Mazzola R, Francolini G, Triggiani L, et al. Metastasis-directed Therapy (SBRT) Guided by PET-CT F-CHOLINE Versus PET-CT Ga-PSMA in Castration-sensitive Oligorecurrent Prostate Cancer: A Comparative Analysis of Effectiveness. *Clin Genitourin Cancer*. 2021;19(3):230-236.

17. Berkovic P, De Meerleer G, Delrue L, et al. Salvage stereotactic body radiotherapy for patients with limited prostate cancer metastases: deferring androgen deprivation therapy. *Clin Genitourin Cancer*. 2013;11(1):27-32.

18. Phillips R, Shi WY, Deek M, et al. Outcomes of Observation vs Stereotactic Ablative Radiation for Oligometastatic Prostate Cancer: The ORIOLE Phase 2 Randomized Clinical Trial. *JAMA Oncol*. 2020;6(5):650-659.

19. Hannan R, Christensen M, Christie A, et al. Stereotactic Ablative Radiation for Systemic Therapy-naïve Oligometastatic Kidney Cancer. *Eur Urol Oncol*. 2022;5(6):695-703.

20. Tang C, Msaouel P, Hara K, et al. Definitive radiotherapy in lieu of systemic therapy for oligometastatic renal cell carcinoma: a single-arm, single-centre, feasibility, phase 2 trial. *Lancet Oncol*. 2021;22(12):1732-1739.

21. Augugliaro M, Marvaso G, Ciardo D, et al. Recurrent oligometastatic transitional cell bladder carcinoma: is there room for radiotherapy? *Neoplasma*. 2019;66(1):160-165.

22. Loi M, Duijm M, Baker S, et al. Stereotactic body radiotherapy for oligometastatic soft tissue sarcoma. *Radiol Med*. 2018;123(11):871-878.

23. Mohamed AA, Goncalves M, Singh BP, et al. Stereotactic radiotherapy in the management of oligometastatic and recurrent head and neck cancer: a single-center experience. *Strahlenther Onkol*. 2024;200(5):400-408.

24. Thariat J, Bosset M, Falcoz A, et al. Survival Without Quality of Life Deterioration in the GORTEC 2014-04 "OMET" Randomized Phase 2 Trial in Patients with Head and Neck Cancer with Oligometastases using Stereotactic Ablative Radiation Therapy (SABR) alone or Chemotherapy and SABR. *Int J Radiat Oncol Biol Phys*. Published online November 28, 2024. doi:10.1016/j.ijrobp.2024.11.084

25. Donovan EK, Lo SS, Beriwal S, et al. Stereotactic Ablative Radiotherapy for Gynecological Oligometastatic and Oligoprogessive Tumors. *JAMA Oncol*. 2024;10(7):941-948.

26. Burkon P, Selingerova I, Slavik M, et al. Stereotactic Body Radiotherapy for Lymph Node Oligometastases: Real-World Evidence From 90 Consecutive Patients. *Front Oncol*. 2020;10:616494.

27. Sogono P, Bressel M, David S, et al. Safety, Efficacy, and Patterns of Failure After Single-Fraction Stereotactic Body Radiation Therapy (SBRT) for Oligometastases. *Int J Radiat Oncol Biol Phys*. 2021;109(3):756-763.

28. Shahi J, Peng J, Donovan E, et al. Overall and chemotherapy-free survival following

© 2025 Willmann J et al. *JAMA Network Open*.

stereotactic body radiation therapy for abdominopelvic oligometastases. *J Med Imaging Radiat Oncol*. 2020;64(4):563-569.

29. Mazzola R, Fersino S, Ferrera G, et al. Stereotactic body radiotherapy for lung oligometastases impacts on systemic treatment-free survival: a cohort study. *Med Oncol*. 2018;35(9):121.

30. Siva S, Sakyanun P, Mai T, et al. Long-Term Outcomes of TROG 13.01 SAFRON II Randomized Trial of Single- Versus Multifraction Stereotactic Ablative Body Radiotherapy for Pulmonary Oligometastases. *J Clin Oncol*. 2023;41(19):3493-3498.

31. Willmann J, Vlaskou Badra E, Adilovic S, et al. Stereotactic body radiotherapy to defer systemic therapy in patients with oligorecurrent disease. *Clin Transl Radiat Oncol*. 2022;37:12-18.

32. Camps-Malea A, Pointreau Y, Chapet S, Calais G, Barillot I. Stereotactic body radiotherapy for mediastinal lymph node with CyberKnife®: Efficacy and toxicity. *Cancer Radiother*. 2023;27(3):225-232.

33. Baker S, Lechner L, Liu M, et al. Upfront Versus Delayed Systemic Therapy in Patients With Oligometastatic Cancer Treated With SABR in the Phase 2 SABR-5 Trial. *Int J Radiat Oncol Biol Phys*. 2024;118(5):1497-1506.

© 2025 Willmann J et al. *JAMA Network Open*.
