# Supplementary material for: Stereotactic Body Radiotherapy Without Systemic Therapy for Oligometastatic Cancer: A Systematic Review and Meta-Analysis
Source: JAMA Netw Open. 2025 Dec 29;8(12):e2549685. doi: 10.1001/jamanetworkopen.2025.49685 (PMC12750244; doi:10.1001/jamanetworkopen.2025.49685)
Supplement: Supplement 2. — Data Sharing Statement [file jamanetwopen-e2549685-s002.pdf]

Data Sharing Statement

Willmann. Stereotactic Body Radiotherapy Without Systemic Therapy for Oligometastatic Cancer. *JAMA Netw Open*. Published December 30, 2025. doi:10.1001/jamanetworkopen.2025.49685

Data
Data available: No
